# Reduced Susceptibility to Vancomycin in *Staphylococcus aureus*

**DOI:** 10.3201/eid1711.110799

**Published:** 2011-11

**Authors:** Marcelo J. Mimica

**Affiliations:** Santa Casa School of Medicine, São Paulo, Brazil

**Keywords:** Staphylococcus aureus, vancomycin, bacteria, antimicrobial resistance, laboratory diagnosis, letter

**To the Editor**: I read with interest the article by Aguado et al. (*1*). I congratulate the authors for their high-quality research and would like to make 2 brief comments.

My first point regards the mechanism by which methicillin-susceptible *Staphylococcus aureus* (MSSA) with reduced susceptibility to vancomycin would also acquire decreased susceptibility to β-lactams. The authors “hypothesize that certain structural modifications might also occur in the cell wall of strains with high vancomycin MIC, including a thicker cell wall as it has been described in MRSA [methicillin-resistant *S. aureus*].” In addition to cell wall thickening, possible mechanisms could include reduction in autolysis (*2**,**3*) and in the cell wall content of penicillin binding protein 4 (PBP4) (*3*). A study of MSSA isolates has shown a reduction in autolysis and in the bactericidal activity of oxacillin after development of intermediate vancomycin susceptibility (*2*). Lower content of PBP4 and decreased autolysis were reported in MRSA isolates after reduced susceptibility to vancomycin developed after exposure to this antimicrobial drug (*3*). Decreased PBP4 has been associated with reduced methicillin susceptibility in *S. aureus* (*4*).

Second, knowing whether the authors had the exact vancomycin MIC of the isolates by broth microdilution to compare with the Etest results would be interesting. In the article, they only state that “all 99 MSSA strains were susceptible to vancomycin (MIC <2 μg/mL) by the broth microdilution method.” Although difficult to compare (because Etest dilution progression is arithmetic and broth microdilution is performed with a geometric dilution), some authors have found substantial differences between the vancomycin MIC results given by these 2 methods (*5*). These differences could have major laboratory and clinical implications.
