# Supplementary material for: Reduced Susceptibility to Vancomycin in Staphylococcus aureus
Source: Emerg Infect Dis. 2011 Nov;17(11):2083–4. doi: 10.3201/eid1711.110799 (PMC3310579; doi:10.3201/eid1711.110799)
Supplement: Related texts

# Reply: Reduced Susceptibility to Vancomycin in *Staphylococcus aureus*

**DOI:** 10.3201/eid1711.111212

**Authors:** Jose Maria Aguado, Rafael San-Juan, Antonio Lalueza, Francisca Sanz, Joaquin Rodríguez-Otero, Carmen Gómez-Gonzalez, Fernando Chaves

**Affiliations:** University Hospital “12 de Octubre,” Madrid, Spain

**In Response:** We appreciate the comments by M. J. Mimica (*1*). We agree that mechanisms other than increased cell wall thickness, such as decreased autolysis, metabolic changes, and reduction in the cell wall content of penicillin binding protein 4, characterize *Staphylococcus aureus* isolates, and they could explain this reduced susceptibility not only to vancomycin but also to β-lactam antimicrobial drugs. A recently published report by Holmes et al. (*2*) confirms this hypothesis. That study showed that *S. aureus* vancomycin MIC >1.5 μg/mL, determined by Etest, was associated with a significantly higher death rate for patients with methicillin-susceptible *S. aureus* bacteremia irrespective of the type of antimicrobial drug used.

Unfortunately, the vancomycin MIC determined by broth microdilution could not be compared with the Etest results because all our isolates had a MIC by broth microdilution that oscillated between 0.5 and 1.0 µg/mL. No isolate had a MIC of 2 µg/mL. In the study by Holmes et al., vancomycin MICs were higher by Etest than by broth microdilution (*1*); however, all isolates were considered vancomycin susceptible by Clinical and Laboratory Standards Institute broth microdilution methods. The authors pointed out that they also found that increased mortality rate was associated with increased broth microdilution MIC (data not shown), although the trend was not so prominent (*2*).

A prospective study would be required to specifically investigate the relationship between vancomycin MIC and outcome of *S. aureus* bacteremia. Our group is performing such a prospective study that we hope will enable us to shed light on this relevant topic.

## References

[1] Mimica MJ. Reduced susceptibility to vancomycin in *Staphylococcus aureus* [letter]. Emerg Infect Dis. 2011;17:2083–4.2209911010.3201/eid1711.110799PMC3310579

[2] Holmes NE, Turnidge JD, Munckhof WJ, Robinson JO, Korman TM, O'Sullivan MV, Antibiotic choice may not explain poorer outcomes in patients with *Staphylococcus aureus* bacteremia and high vancomycin minimum inhibitory concentrations. J Infect Dis. 2011;204:340–7. 10.1093/infdis/jir27021742831
